# Effect of supplementation with *n*-3 polyunsaturated fatty acids and/or β-glucans on performance, feeding behaviour and immune status of Holstein Friesian bull calves during the pre- and post-weaning periods

**DOI:** 10.1186/s40104-019-0317-x

**Published:** 2019-01-29

**Authors:** Ruairi P. McDonnell, John V. O’ Doherty, Bernadette Earley, Anne Marie Clarke, David A. Kenny

**Affiliations:** 1 School of Agriculture, Food Science and Veterinary Medicine, University College Dublin, Belfield, Dublin 4, Ireland; 2 Teagasc Animal and Bioscience Research Department, Animal and Grassland Research and Innovation Centre (AGRIC), Grange, Dunsany, Co. Meath, Ireland; 3 Present address: GippsDairy, 71 Korumburra-Warragul Road, Warragul, VIC 3820 Australia

**Keywords:** Fish oil, Fucoidan, Immune function, Laminarin, Preweaning, Seaweed

## Abstract

**Background:**

Previous research in both calves and other species has suggested *n*-3 polyunsaturated fatty acids (PUFA) and β-glucans may have positive effects on immune function. This experiment measured performance, behaviour, metabolite and immunological responses to pre-weaning supplementation of dairy bull calves with *n*-3 PUFA in the form of fish oil and β-glucans derived from seaweed extract. 44 Holstein Friesian bull calves, aged 13.7 ± 2.5 d and weighing 48.0 ± 5.8 kg were artificially reared using an electronic feeding system. Each calf was offered 5 L (120 g/L) per day of milk replacer (MR) and assigned to one of four treatments included in the MR, (1) Control (CON); (2) 40 g *n*-3 PUFA per day (FO); (3) 1 g β-glucans per day (GL) and (4) 40 g *n*-3 PUFA per day & 1 g/d β-glucans (FOGL) in a 2 × 2 factorial design. Milk replacer and concentrate was offered from d 0–62 (pre-weaning), while concentrate provision continued for a further 31 d post-weaning period. Individual daily feed intake and feeding behaviour was recorded throughout, while bodyweight and blood analyte data were collected at regular intervals.

**Results:**

Overall mean concentrate DMI from d 0–93 was 1.39, 1.27, 1.00 and 0.72 kg/d for CON, FO, GL and FOGL calves, respectively (SEM = 0.037; *P* < 0.0001). Calves supplemented with GL were significantly lighter (*P* < 0.0001) at both weaning (d 62) and turnout to pasture (d 93) than un-supplemented calves, with a similar effect (*P* < 0.0001) evident for calves receiving FO compared to un-supplemented contemporaries. Supplementation with GL reduced the number of unrewarded visits where milk was not consumed (*P* < 0.0001) while supplementation with FO increased mean drinking speed (*P* < 0.0001). Supplementation with GL resulted in greater concentrations of haptoglobin (*P* = 0.034), greater serum osmolality (*P* = 0.021) and lower lymphocyte levels (*P* = 0.027). In addition, cells from GL supplemented calves exhibited a lower response than un-supplemented contemporaries to both Phytohaemagglutinin A stimulated IFN-γ (*P* = 0.019) and Concanavalin A stimulated IFN-γ (*P* = 0.012) following in vitro challenges.

**Conclusions:**

Pre-weaning supplementation of bull calves with either *n*-3 PUFA or β-glucan resulted in reduced voluntary feed intake of concentrate and consequently poorer pre-weaning calf performance. There was no evidence for any beneficial effect of either supplementation strategy on calves’ immune responses.

## Background

Neonatal mortality represents a significant economic loss in dairy production systems worldwide. Raboisson et al. [1] reported a calf mortality rate on French dairy farms of 5.7% in calves aged from 3 d to 1 month, while in the USA, pre- and post-weaned calf and heifer losses have been estimated at 9.6% per annum, with pre-weaned heifer calves accounting for 82% of these losses [2]. Prohibition on the use of antimicrobial agents and antibiotic growth promoters in animal feed in the European Union has necessitated the development of alternative strategies to augment immune function in farm animals [3]. Consequently, interest has grown over recent years in the potential of dietary additives as a means to enhance the immune response of dairy calves. For example, there is some evidence for non-ruminants that consumption of certain polyunsaturated fatty acids (PUFA) belonging to the omega-3 (*n*-3) fatty acid series, including eicosapentaenoic acid (EPA; 20:5 *n*-3), docosapentaenoic acid (22:5 *n-*3) and docosahexaenoic acid (DHA; 22:6 *n*-3), can affect immune function [4]. Earlier research showed how dietary supplementation with EPA and DHA enriched fish oil (FO) resulted in supressed proliferation of T-lymphocytes (and in some cases B-lymphocytes) in a number of species, compared with other forms of dietary fat such as lard, corn oil, linseed oil and hydrogenated coconut oil [5]. A review by Calder [6] summarised how EPA and DHA can inhibit various aspects of inflammation including leukocyte chemotaxis, production of certain eicosanoids, and inflammatory cytokines, ultimately leading to altered expression of inflammatory genes.

Both positive and negative effects of *n*-3 PUFA supplementation have been reported in livestock species depending on the specific fatty acid and the dietary inclusion rate. For example, Ballou and DePeters [7] showed that *n*-3 PUFA supplementation of milk replacer (MR) in pre-weaned Jersey calves altered the phagocytic function of monocytes and the oxidative burst capacity of neutrophils, indicating that it may be possible to positively influence immune function. Supplementation with a FO supplement rich in *n*-3 PUFA was also shown to potentiate the immune response to nematode parasite infection in calves [8]. Onset of septicaemia in calves, from which survival rates are low, is generally preceded by an over-aggressive inflammatory response [7]. Two published studies have reported a reduced inflammatory response across different species offered diets supplemented with *n*-3 PUFA [4, 9]. Furthermore, Jersey bull calves consuming 1.5, 2.8 and 4.1 g *n*-3 PUFA/100 g FA, challenged with *Salmonella* Typhimurium lipopolysaccharide, exhibited a reduced respiratory rate in the first 6 h with increasing levels of *n*-3 PUFA [10], which the authors suggested may be beneficial in preventing an excessive acute phase response.

Carbohydrate based derivatives containing β-glucans (GL) have likewise been shown to augment immune function in several species [3]. However, significant variation in the solubility and biochemical characteristics of GL from different sources exists and this factor has been proven to influence gene expression of various aspects of immune function in non-ruminants, including expression of pro- and anti-inflammatory cytokine markers [11]. Laminarin is a seaweed derived GL, with varying chemical structures depending on whether it is derived from *L. digitata* or *L. hyperborea* species, while GL derived from yeast (*Saccharomyces cerevisae*) differ yet again in chemical structure from *Laminaria* species [11]. Previously Reilly et al. [12] reported a marginal effect on immune response in weaned pigs supplemented with seaweed extract containing laminarins and fucoidans. Laminarin derived from *L. digitata* contains water soluble GL rich in β-(1,6) linked side chains. These GL are believed to stimulate the host immune system by promoting the production of cytokines and chemokines and also activate leukocytes such as macrophages and neutrophils [13, 14]. Leonard et al. [4] observed a number of immunocompetence related differences in piglets suckling sows consuming *L. digitata* derived seaweed extract, including elevated circulatory immunoglobulin G concentrations early in lactation, thus enhancing humoral immune function, as well as decreased eosinophil numbers and increased leukocyte phagocytosis. However, published studies investigating the effects of GL supplementation on neonatal calves have mainly used yeast extract containing *Saccharomyces cerevisiae.* Eicher et al. [15] detailed how supplementation with two contrastingly purified forms of GL derivative from yeast cell walls mixed with MR, altered feed intake, the presence of *Escherechia coli* O157 and leukocyte function in calves subjected to a transport stressor. A recent study reported marginally increased starter intake in the first month of life and greater pre-weaned average daily gain (ADG) in high-risk Holstein bull calves supplemented with 1 g/d of GL, compared to control calves [16], while Kim et al. [17] observed increased production of immune-related serum proteins and positive haematological prognostic indicators, as well as improved general health conditions in the calves supplemented with hydrolysed yeast containing GL following a vaccine challenge.

To date, however, the cumulative or interactive effects of dietary supplementation with *n*-3 PUFA and GL extracted from seaweed to the diets of pre-weaned calves, and subsequent effects on aspects of immune function have not, to our knowledge, been reported on. The objective of this experiment was to evaluate the effect of supplementation with long chain *n*-3 PUFA and/or GL on performance, behaviour and immune status of artificially reared Holstein-Friesian (HF) bull calves during the pre- and post-weaning periods.

## Methods

### Experimental design

Forty-four HF male calves were used in this experiment. Calves were sourced from 30 different farms across 3 geographical regions and were transported to the research facility at approximately 12 days of age. Mean (± SD) age and bodyweight at the start of the experiment were 13.7 ± 2.5 d and 48.0 ± 5.8 kg, respectively. The experiment was structured as a 2 × 2 factorial design, and calves were blocked by bodyweight, age and source region, before random allocation within block to one of four treatments: (1) Control (CON); (2) 40 g *n*-3 PUFA/d (FO); (3) 1 g β-glucans/d (GL) and (4) 40 g *n*-3 PUFA/d & 1 g β-glucans/d (FOGL). Dosage rates of GL were based on research from our group on weaned pigs [11, 12], scaled to the bodyweight of pre-weaned HF calves. Previous studies from ourselves [18] and others [7, 10] have indicated that approx. 2% of DM is close to the upper limit of FO supplementation possible, without overly affecting DMI. Therefore 40 g/d of FO was selected based on 2% of the expected average pre-weaning DMI of HF calves. For convenience, calves that were supplemented with FO are referred to as FO+, while calves that did not receive any FO supplement are denoted as FO–. Similarly calves that were supplemented with GL are referred to as GL+ and calves that received no GL supplement are denoted as GL–. The β-glucan supplement (1 g) contained laminarin (0.10 g), fucoidan (0.08 g) and ash (0.82 g) and was extracted from seaweed containing *Laminaria* spp. as described by [19]. The seaweed extract was obtained from a commercial company (Bioatlantis Ltd., Tralee, Co. Kerry, Ireland). The *n*-3 PUFA were provided in the form of FO, which was also obtained from a commercial company (Trouw Nutrition, Belfast, UK), and derived from anchovy, sardine and salmon oil; however the oil was distilled to concentrate the EPA and DHA content. This novel concentrated FO supplement was chosen as a means to supply the PUFA over other options such as flaxseed oil due to our belief that it was the most potent method commercially available to us to enrich tissue and systemic concentrations of PUFA in the calves, plus we had successfully used this product in other published studies prior to the experiment [4, 18]. Calves that did not receive FO were supplemented with a fixed amount of soya oil (SO) in order to ensure all diets were iso-lipidic. All diets were also effectively isocaloric as each calf received 1.48 MJ of gross energy (GE) daily as either FO or SO, and the daily GL supplement only contained 0.016 MJ of GE. The fatty acid composition of the concentrated FO is presented in Table 1. Calves were only fed the treatments during the pre-weaning period and did not receive any FO or GL once weaned off milk replacer (MR).

**Table 1:** Fatty acid composition of fish oil (% of total fatty acids)

Fatty acid	Fish oil (*n* = 4)
C14:0	0.14 ± 0.01
C15:1	0.14 ± 0.09
C16:0	2.27 ± 0.35
C16:1	1.04 ± 0.06
C17:0	0.19 ± 0.07
C17:1	0.07 ± 0.01
C18:0	3.64 ± 0.21
C18:1*n*-9 *cis*	5.70 ± 0.45
C18:1*n*-9 *trans*	0.08 ± 0.01
C18:2*n*-6	1.54 ± 0.02
C18:3*n*-3	0.89 ± 0.04
C18:3*n*-6	0.19 ± 0.02
C20:1	3.36 ± 0.10
C20:2	3.00 ± 0.22
C20:3*n*-3	0.32 ± 0.03
C20:3*n*-6	0.43 ± 0.04
C20:4*n*-6	1.98 ± 0.09
C20:5*n*-3	39.05 ± 0.54
C22:0	0.50 ± 0.04
C22:1*n*-9	1.31 ± 0.32
C22:2	2.37 ± 0.04
C22:6*n*-3	24.25 ± 0.14
C24:0	0.47 ± 0.09
C24:1	0.28 ± 0.02

### Animal management

After an initial acclimatisation period (1–2 d), calves were group penned on barley straw bedding according to age to minimise the transfer of disease between animals, and a space allocation of in excess of 3 m^2^ per calf (approximately twice European Union guidelines) was provided with *ad libitum* access to fresh drinking water. Calves were fed an industry standard MR (Blossom Easymix, Volac, Co. Cavan, Ireland; Table 2) using a computerised feeder (Forster-Technik SA 2000, Engen, Germany), and offered *ad libitum* access to concentrates and a limited amount of hay for 62 d. Concentrate intake was also measured daily using the computerised feeder. This period is referred to as the pre-weaning period. For all four treatments, water was heated to approximately 42^o^ C and 120 g of MR was added per litre of water. Calves received their respective supplement as per treatments above, via specialised dispensers for liquid additives (Forster-Technik SA 2000, Engen, Germany), which were calibrated twice weekly. Calves were allowed access to a maximum of five litres of MR per day in two allowances of 2.5 L for the duration of the pre-weaning period (gradually increased from 2 L to 5 L over 5 d at the start of the experimental period). The mean ambient temperature inside the calf shed (logged every 15 min) was 11.9 ± 4.9 °C (range: − 0.6 to 30.9^o^ C).

**Table 2:** Chemical composition of concentrates and calf milk replacer

Item	Concentrates	Milk replacer
DM, g/kg	912	–
Crude protein, g/kg DM	181	228
Ash, g/kg DM	66	–
ADF, g/kg DM^a^	136	–
NDF, g/kg DM^b^	407	–
Gross energy, MJ/kg DM	168	203
Percentage oil, g/kg DM	21	161

^a^*ADF* Acid detergent fibre

^b^*NDF* Neutral detergent fibre

From d 55 calves were gradually weaned off MR over the following 7 d, and were only fully weaned when consuming a minimum of 1 kg/d of concentrate for three consecutive days. The period from d 62–93 is referred to as the post-weaning period. Following turn-out to pasture (d 94), calves grazed together in a paddock-based rotational grazing system for 80 d (post-turnout period). All performance and intake data is presented relative to these three periods. However, for blood hormone, metabolite and haematology variables, data are presented relative to three different periods, the pre-weaning period (d 0–61), the peri-weaning period (immediately after weaning; d 62–70) and the post-weaning period (d 71–93). Calves continued to be offered free access to concentrates, water, and a limited amount of hay from d 62–93, before turnout to pasture.

### Animal measurements

#### Feed intake and growth

Individual milk and concentrate consumption was recorded in the pre-weaning period while post-weaning concentrate consumption was recorded until d 93 when calves were turned out to pasture. Consumption of hay was not measured in this study and was assumed to contribute very minimally to overall calf nutrition. Calves were weighed in the morning, at 7 d intervals using calibrated electronic scales, resulting in eight and five weight records per calf during the pre-weaning and post-weaning periods, respectively. Three weight records per calf were recorded at approximately 28 d intervals while grazing pasture during the post-turnout period.

#### Feeding behaviour and fecal scoring

Feeding behaviour traits were evaluated using Kalb Manager software (Forster Technik SA 2000, Engen, Germany). Daily MR and concentrate feeding events were calculated as the number of occasions a calf entered the milk or concentrate feeding stalls and consumed a minimum of 100 mL of MR or 10 g of concentrate, respectively. Non-feeding events for MR and concentrate were calculated as the number of times a calf entered the stall and consumed ≤100 mL and ≤ 10 g respectively. Drinking speed was also recorded (L/min) for each feeding event, in addition to the cumulative number of minutes each calf spent in the concentrate feeding stalls.

Fecal scores and rectal temperatures were recorded biweekly for five consecutive days during the pre-weaning period, and weekly for three to five consecutive days from d 55–69. Fecal consistency was scored by the same experienced technician for the duration of the experiment, using the following scale: 1 = firm; 2 = semi-solid; 3 = liquid and 4 = very liquid [20]. Body temperatures (degrees Celsius; °C) were also recorded biweekly using a digital thermometer in the morning prior to the first allocation of feed each day. Body temperature and fecal scores were recorded in the morning before feeding, so as not to confound the interpretation of body temperature data.

#### Blood collection and analysis

Blood was collected from all calves by jugular venepuncture using mild restraint in a holding chute on d 0, 29 & 55 (pre-weaning), d 62, 63, 65 and 70 (peri-weaning), and d 76 and 93 (post-weaning) of the experimental period. Blood was collected into evacuated vials (Vacuette, Cruinn Diagnostics, Ireland) containing the appropriate anticoagulants for subsequent haematology and clinical biochemical analysis. Samples were immediately transported to the laboratory upon completion of sampling in iced water, stored at ambient temperature and processed within 3.5 h. Blood samples collected into lithium heparinized vacutainers (9 mL) were used to determine the concentrations of β-hydroxybutyrate (βHBA), haptoglobin, and urea. Sodium fluoride anti-coagulated blood (4 ml) was used to determine the concentration of glucose. Plasma was harvested following centrifugation at 1,600×*g* at 4 °C for 15 min and samples were stored at − 20 °C until assayed. Plasma glucose, urea and βHBA concentrations were analysed on an automatic analyser (Olympus AU400, Japan) using reagents supplied by Olympus. Concentration of plasma haptoglobin was measured using an automatic analyser (SPACE, Alfa Wassermann, Inc., West Caldwell, NJ, USA) and commercial assay kit (Tridelta Development Ltd., Wicklow, Ireland) according to the manufacturer’s procedure [21]. Total leukocyte, neutrophil, lymphocyte, and monocyte numbers were determined from blood vials (6 mL) containing K_3_EDTA anti-coagulant using an automated haematology analyser (AV ADIVA 2120, Bayer Healthcare, Siemens, UK) equipped with software for bovine blood. A whole blood culture procedure [22] was used to determine the in vitro lymphocyte production of interferon gamma (IFN-γ) in lithium heparinised whole blood. Duplicate 1.48 mL aliquots of blood were cultured in sterile 24-well flat culture plates (Sarstedt Ltd., Drinagh, Wexford, Ireland) with 20 μL of PBS (GibcoBRL, Life Technologies Ltd., Paisley, Scotland, UK) containing 1.0 mg/mL of concanavalin A (Con A; Sigma-Aldrich, Inc., UK), 1.0 mg/mL phytohaemagglutinin (PHA; Sigma-Aldrich, Inc., UK) or no additive, for 24 h at 37 °C and in an atmosphere of 5% CO_2_. The culture plates were then centrifuged at 1,600×*g* at 4 °C for 20 min, supernatant harvested and frozen at − 20 °C until assayed for INF-γ using an ELISA procedure specific for bovine plasma (BOVIGAM, Biocor Animal Health, NE, USA), as previously described by [23]. The in vitro Con A or PHA stimulated lymphocyte production of IFN-γ was calculated by subtracting the absorbance at 450 nm of wells that received PBS alone from wells that received Con A or PHA, respectively. Serum osmolality was measured with a veterinary refractometer (DLC Australia Pty Ltd., Caboolture, QLD, 4510 Australia).

Plasma concentrations of insulin-like growth factor 1 (IGF-1) were quantified using radio-immuno assay following an acid ethanol extraction. Intra-assay coefficients of variation for IGF-1 samples were 33.2%, 21.7% and 24.4% for low, medium and high standards, respectively, while inter-assay coefficients of variation were 50.1%, 14.1% and 12.6% for corresponding low, medium and high standards. Plasma concentrations of insulin were quantified using Coat-a-Count Insulin RIA assay (Siemens, LA, USA). Intra-assay coefficients of variation for insulin were 16.8%, 13.8% and 10.2% for the low, medium and high standards, respectively, while inter-assay coefficients of variation for insulin were 8.4%, 6.0% and 3.8% for corresponding low, medium and high standards. The fatty acid (FA) content of plasma was determined as described [18], at two different timepoints (d 0 and d 29). Plasma FA analysis was only carried out for six calves in the CON group, seven in the FO group, six in the GL group and eight in the FOGL group.

### Feed analysis and trait derivations

The FA composition of the FO was analysed by gas chromatography [24]. Samples of the concentrate pellets were taken twice weekly, and composited into weekly samples, before storage at − 20 °C pending analysis for DM, crude protein, neutral detergent fibre, acid detergent fibre, ash, gross energy (GE) and percentage oil. Samples were milled through a 1-mm screen using a Christy and Norris hammer mill (Christy and Norris Process Engineers Ltd., Chelmsford, UK); DM was determined by oven drying at 104 °C for a minimum of 16 h. Ash was determined on all materials after ignition of a known weight of ground material in a muffle furnace (Nabertherm, Bremen, Germany) at 550 °C for 4 h. The neutral and acid detergent fibre concentrations of the concentrate were obtained using an Ankom-200 fiber analyzer (Ankom Technology, Fairport, NY) [25]. The crude protein content (total N × 6.25) was determined with a Leco FP 528 nitrogen analyzer (Leco Instruments UK Ltd., Newby Road, Hazel Grove, Stockport, Cheshire, UK) [26]. Ether extract was determined with a Soxtec instrument (Tecator, Hoganas, Sweden), while GE was determined with a Parr 1201 oxygen bomb calorimeter (Parr, Moline IL).

Body weight gain was calculated by fitting a linear regression through body weights recorded during the experiment.

### Statistical analysis

All data analysis was conducted using appropriate procedures of the Statistical Analysis Systems software v9.1 (SAS Institute, Cary, NC, USA). Data were tested for normality of distribution (UNIVARIATE procedure) and, where appropriate, transformed to the power of lambda (TRANSREG procedure). Data were then subjected to a repeated measures ANOVA (MIXED procedure). Differences in individual least-square means were evaluated using the Tukey-Kramer adjustment. Diet (FO vs GL), sample day (or period), block, and their interactions were included in the model as fixed effects. Calf age (range of 9 d) was included as a covariate. Neutrophil to lymphocyte ratio, measured on blood samples collected on the day of allocation to treatment was used as a proxy for immune status and was also initially included as a co-variate in the statistical analysis, as appropriate. If not statistically significant (*P* > 0.05), co-variates and interaction terms were subsequently excluded from the final model. Animal was treated as a random effect, while sample day or period were treated as a repeated effect for all analyses. Treatment effects on animal performance, feed intake and feeding behaviour and all blood analyte data (metabolic hormones, metabolites, haematology variables and fatty acids) were determined using mixed models ANOVA and specifically the MIXED procedure with the REPEATED statement employed where appropriate.

## Results

### Feed intake

No three-way interactions were identified between FO, GL and period (*P* > 0.05) for any feed related variable measured (Table 3). No main effects of treatment on MR intake were detected, however a FO × GL interaction (*P* = 0.04) was observed but not considered to be of biological importance, with the greatest differences in mean MR intake between all four treatments still less than 0.1 L/d (mean MR intake 4.69, 4.68, 4.65 and 4.63 L/d for CON, FO, GL and FOGL, respectively).

**Table 3:** Effect of FO and GL supplementation on feed intake in the pre- and post-weaning periods

Trait	Fish Oil (FO)			β -Glucans (GL)			Period (P) relative to weaning				*P*-value		Interactions^b^		
	–	+	SED	–	+	SED	Pre	Post	SED	FO	GL	P	FO×GL	P×FO	P×GL
Milk replacer, litres/d	4.65	4.66	0.034	4.67	4.64	0.034	4.66	n/a	n/a	0.80	0.45	n/a	0.038	n/a	n/a
Concentrates, kg DM/d	1.20	0.99	0.018	1.32	0.86	0.019	0.61	1.58	0.026	<.0001	<.0001	<.0001	0.0013	ns	<.0001
Total GEI, MJ/d^a^	25.44	21.96	0.307	27.36	20.03	1.714	21.62	25.78	0.510	<.0001	<.0001	<.0001	0.0082	0.02	<.0001

^a^Total Gross Energy Intake –Includes gross energy of FO (37 MJ/kg) and/or GL (16 MJ/kg) where appropriate

^b^No FO×GL×P interactions were detected. Interactions are described in the text when significant

*ns* not significant, *n/a* not applicable

A dietary treatment interaction was observed for concentrate intake, which averaged 1.39, 1.27, 1.00 and 0.72 kg/d for CON, FO only, GL only and FOGL calves, respectively (SEM = 0.037; *P* < 0.0001). In addition, a period × GL interaction for concentrate intake was manifested as a greater difference in concentrate intake between non-GL supplemented calves and GL supplemented calves in the post-weaning period (1.97 vs 1.19 kg/d respectively; SED = 0.037), compared to pre-weaning (0.69 vs 0.53 kg/d respectively; SED = 0.026).

These results were reflected in the GE intake (GEI) data, where a FO × GL interaction was also detected, with mean GEI values of 28.6, 26.1, 22.2 and 17.8 MJ/d observed for CON, FO, GL and FOGL calves, respectively (SEM = 0.42; *P* < 0.0001). Period × GL and period × FO interactions for GEI were also observed. Similar to concentrate intake, the difference in GEI between non-GL and GL supplemented calves was greater in the post-weaning period (32.08 vs 19.47 MJ/d respectively; SED = 0.612) than the pre-weaning period (22.64 vs 20.60 MJ/d respectively; SED = 0.391). The period × FO interaction for GEI was also manifested as a greater difference between non-FO and FO supplemented calves post-weaning (28.0 vs 23.56 MJ/d; SED = 0.611) than in the pre-weaning period (22.88 vs 20.36 MJ/d; SED = 0.390).

### Feeding behaviour

The effect of calf MR supplementation with FO and GL on feeding behaviour is summarised in Table 4. No difference (*P* > 0.05) was detected between FO supplemented or un-supplemented calves in the number of visits to the MR feeder per day where feed was or was not consumed. There was also no difference (*P* > 0.05) in the number of visits per day where milk was consumed between GL supplemented and un-supplemented calves. However GL supplemented calves had less visits to the feeder where milk was not consumed (*P* = 0.02) than un-supplemented calves. Drinking speed was greater (*P* = 0.02) for FO supplemented than un-supplemented calves, but did not differ between GL supplemented and un-supplemented calves (*P* = 0.85).

**Table 4:** Effect of fish oil and β-glucans supplementation in calf milk replacer on feeding behaviour

No. of visits per day where:	Fish Oil (FO)			β-Glucans (GL)			Period (P) relative to weaning			*P*-value^a^						
	–	+	SED	–	+	SED	Pre	Post	SED	FO	GL	P	FO×GL	FO×P	GL×P	FO×GL×P
Milk consumed	3.45	3.62	0.164	3.50	3.57	0.164	–	–	–	0.29	0.68	–	ns	–	–	–
Milk not consumed	11.85	11.66	1.102	13.08	10.44	1.103	–	–	–	0.87	0.02	–	ns	–	–	–
Drinking rate, L/min	1.09	1.21	0.053	1.15	1.15	0.053	–	–	–	0.02	0.85	–	ns	–	–	–
Visits where concentrate was consumed, visits/d	19.38	20.42	1.798	21.61	18.20	1.798	20.84	18.97	0.638	0.29	0.056	0.003	0.071	<.0001	ns	<.0001
Time in concentrate feeder, min/d	55.40	51.45	2.894	56.99	49.86	2.893	39.56	67.29	1.091	0.17	0.014	<.0001	ns	<.0001	ns	ns

^a^Interactions are described in the text when significant

*ns* not significant

A three way interaction (*P* < 0.0001) was observed between FO, GL and period in the number of visits to the concentrate feeder, which was manifested as a change in rank between the two periods. In the pre-weaning period the daily number of visits per calf to the concentrate feeder was 22.9, 22.4, 20.9 and 17.2 (SEM = 1.83) for CON, FO only, GL only and FOGL calves, respectively, while during the post-weaning period the daily number of visits to the concentrate feeder was 25.1, 17.7, 17.0 and 16.1 (SEM = 1.86) for FO only, GL only, FOGL and CON calves, respectively. A FO × period interaction was also observed for cumulative min/d spent in the concentrate feeder (*P* < 0.0001) whereby during the pre-weaning period, FO supplemented calves spent less time in the concentrate feeder compared to non-FO supplemented calves (35.27 vs 43.84 min/d respectively; SED = 2.933), with no difference observed post-weaning.

### Performance, fecal scores and rectal temperatures

The effects of dietary FO and GL supplementation on calf performance, fecal scores and rectal temperatures in the periods relative to weaning are summarised in Table 5. No three-way interactions were detected for any of the variables presented (*P* > 0.05). There was an interaction between FO and GL for end weight (*P* = 0.04) whereby FO only, GL only, FOGL and CON calves, respectively weighed 156, 156, 149 and 169 kg (SEM = 1.46). We also observed a FO × period interaction for ADG (Table 5), where during the pre-weaning period, FO supplemented calves had lower ADG than non FO supplemented calves (0.24 vs 0.38 kg/d; SED = 0.043; *P* = 0.013 for FO+ vs FO–), while no effects of FO supplementation in the post-weaning and post turnout periods were shown. A further interaction was detected between GL and period for ADG (Table 5), whereby no effect of GL supplementation was observed in the pre-weaning and post-turnout periods, while during the post-weaning period, GL supplemented calves had lower mean ADG than non GL supplemented calves (1.00 vs 1.28 kg/d; SED = 0.044; *P* < 0.0001). During the post-weaning period, animals supplemented with FO had an ADG of 1.22 which was greater than the FOGL group (1.22 vs 0.91 kg/d; SEM = 0.88; *P* = 0.029). The CON animals also had a greater ADG than the FOGL group during this period (1.34 vs 0.91 kg/d; SEM = 0.86; *P* = 0.002). Calf ADG was greatest during the post-weaning period when concentrates were the main dietary component.

**Table 5:** Effect of FO and GL supplementation on animal performance, rectal temperature and fecal scores

	Fish Oil (FO)			β-Glucans (GL)			Period (P) relative to weaning				*P*-value^f^					
	–	+	SED	–	+	SED	Pre	Post	Post-turnout	SEM	FO	GL	P	FO×GL	FO×P	GL×P
Start weight, kg	48.7	48.1	0.43	48.5	48.3	0.42					ns	ns		ns	ns	ns
Weaning weight, kg^a^	66.1	60.0	0.39	65.2	60.9	0.39					<.0001	<.0001		ns	ns	ns
Turnout weight, kg^b^	108.4	95.8	0.95	110.0	94.1	0.95					<.0001	<.0001		ns	ns	ns
End weight, kg^c^	162.4	152.6	1.48	162.4	152.6	1.45					<.0001	<.0001		0.04	ns	ns
ADG, kg/d^d^	0.78	0.71	0.042	0.81	0.68	0.042	0.31	1.14	0.78	0.022	0.060	0.001	<.0001	ns	<.0001	<.0001
Fecal score^e^	2.15	2.15	0.080	2.1	2.2	0.08	2.06	2.24		0.056	0.984	0.098	0.002	ns	ns	ns
Rectal temperature, **°**C	38.86	38.63	0.057	38.69	38.81	0.057	38.69	38.81		0.046	< 0.0001	0.035	0.011	ns	ns	ns

^a^Weaning weight = weight at end of milk feeding period

^b^Turnout weight = weight at end of concentrate feeding and before turn out to grass

^c^End weight = weight after 3 months at grass

^d^ADG = Average daily gain over a 172-day period starting on d 0 of the experiment

^e^Fecal score ranged from 1 to 4; 1 = firm, 2 = semi-solid, 3 = liquid, 4 = very liquid

^f^Interactions are described in the text when significant

*ns* not significant

Calves on the FO– and GL– diets had greater (*P* < 0.001) weaning, turnout and end weights than calves on the FO+ and GL+ diets. No effect (*P* > 0.05) of FO was observed in fecal scores, however GL supplemented calves tended to have greater fecal scores than non GL supplemented calves (*P* = 0.098). Fecal scores for all calves were lower (*P* = 0.002) in the pre-weaning period than the post-weaning period. Rectal temperatures were found to be generally within the normal range for all calves but were lower (*P* < 0.001) for calves supplemented with FO compared to non-FO supplemented calves. In contrast, for GL, un-supplemented calves had lower (*P* = 0.035) mean rectal temperatures than supplemented calves. Lower rectal temperatures in all calves were observed in the pre-weaning period than the post-weaning period (*P* = 0.011).

### Plasma fatty acid content

Table 6 shows the mean concentration of fatty acid methyl esters (FAME; as a % of total FAME) in plasma at two timepoints, (1) before the experiment started and (2) while MR was being supplemented with FO, GL and soya oil (d 29). There was a three way FO × GL × timing interaction for C16:0 whereby in timing 2, FOGL calves had the highest content of C16:0 (28.9%) compared to calves receiving FO only or CON (21.4% and 23.1% respectively), with GL only calves lowest at 16.9%. A similar three way interaction was detected for C18:0, whereby no effect of treatment was observed in timing 1, while in timing 2 CON calves had a greater (*P* = 0.039) concentration of C18:0 (13.58%) than FO only, GL only and FOGL calves which did not differ (9.21%, 8.75% and 10.73% respectively).

**Table 6:** Effect of FO and GL supplementation on plasma concentrations of selected fatty acid methyl esters (FAME) as a % of total FAME

	Fish oil (FO)			β-Glucans (GL)			Timing^c^				*P*-value^a^				
	–	+	SED	–	+	SED	1	2	SED	FO	GL	T	FO × GL	FO × T	FO × GL × T
C16	15.1	20.5	1.70	18.2	17.5	1.70	13.0	22.6	1.70	0.003	0.684	<.0001	0.074	ns	0.0321
C16:1	16.7	14.7	1.40	14.9	16.5	1.36	22.2	9.2	1.50	0.168	0.257	<.0001	ns	ns	ns
C18	8.0	8.2	0.68	8.6	7.7	0.68	5.7	10.6	0.68	0.817	0.193	<.0001	0.015	0.053	0.0388
C18:1*n*-9c	7.3	6.8	0.57	7.2	7.0	0.57	6.8	7.3	0.57	0.459	0.721	0.442	ns	ns	ns
C18:2*n*-6c	22.0	12.0	1.09	18.2	15.9	1.09	7.9	26.2	1.09	<.0001	0.038	<.0001	ns	<.0001	ns
C20 + C20:1	1.3	1.5	0.20	1.3	1.5	0.21	1.8	1.0	0.21	0.203	0.240	0.001	ns	ns	ns
C22:1*n*-9c + 20:3*n*-6	1.0	1.4	0.22	1.1	1.2	0.21	1.2	1.1	0.22	0.128	0.668	0.612	ns	0.035	ns
EPA	0.938	5.616	1.02	4.056	5.617	0.807	nd^b^	7.2	2.94	0.0003	0.166	<.0001	ns	<.0001	ns
DHA	0.010	0.830	0.05	1.045	0.401	0.34	nd	1.1	–	<.0001	0.338	–	ns	0.001	ns

^a^No significant GL × Timing interactions observed (*P* > 0.05). Interactions are described in the text when significant

^b^Non detectable

^c^Blood sample timing; Timing 1 = d 0 (pre-supplementation), Timing 2 = d 29

There was a FO × timing interaction for C18:2*n*-6c (linoleic acid), whereby no difference in FO+ and FO– calves was detected in timing 1, while in timing 2, FO+ calves had lower concentrations of C:18:2*n*-6c than FO- calves (17.38% vs 34.94%; SED = 1.545, *P* < 0.001). An additional FO × timing interaction was observed for C22:1*n*-9c + C20:3*n*-6, whereby FO+ calves had greater levels than FO– calves in timing 2 only (1.58% vs 0.68%, SED = 0.342, *P* = 0.024). There were also FO × timing interactions observed for the *n*-3 PUFA C20:5*n*-3 (EPA) and C22:6*n*-3 (DHA). No EPA or DHA was detected in timing 1, however in timing 2, EPA was greater in FO+ calves than FO– calves (12.25% vs 2.19%, SED = 2.93, *P* = 0.004). Likewise DHA was greater in FO+ calves than FO– calves in timing 2 (*P* = 0.0025).

### Blood hormones and metabolites

The effects of supplementation of calves with FO and GL as well as period effects on systemic hormones and metabolites are summarised in Table 7. No three-way interactions were detected between the main effects for any plasma analyte measured (*P* > 0.05). There was a FO × GL interaction for plasma insulin (*P* < 0.0064) whereby CON calves had greatest concentrations of plasma insulin, with GL calves also having greater levels than FO and FOGL calves (mean plasma insulin concentration = 3.15, 2.15, 1.48 and 1.64 μIU/mL for CON, GL, FO and FOGL calves, respectively; SEM = 0.370). In addition, there was a FO × period interaction for insulin (*P* = 0.037), which was manifested as no effect of FO supplementation in the pre-weaning or post-weaning periods, while during the peri-weaning period FO supplemented calves had lower (*P* = 0.006) insulin concentrations (0.65 vs 1.98 μIU/mL; SED = 0.246 for FO+ vs FO–).

**Table 7:** Effect of FO and GL supplementation on plasma concentrations of metabolic hormones and metabolites

	Fish Oil (FO)			β-Glucans (GL)			Period (P) relative to weaning				*P*-value^a^					
	–	+	SED	–	+	SED	Pre	Peri	Post	SED	FO	GL	P	FO×GL	FO×P	GL×P
Hormones																
Insulin, μIU/mL	2.65	1.56	0.197	2.31	1.89	0.197	2.59	1.31	2.40	0.311	0.005	0.338	<.0001	0.0064	0.037	ns
IGF, ng/mL	107.56	66.98	16.091	103.77	70.77	16.087	79.37	62.04	120.40	10.368	0.012	0.173	<.0001	ns	0.057	<.0001
Metabolites, mmol/L																
Glucose	4.00	3.55	0.139	3.99	3.55	0.139	3.78	3.57	3.97	0.139	0.001	0.002	0.0001	ns	ns	0.058
ΒHBA	0.27	0.27	0.018	0.26	0.29	0.018	0.12	0.31	0.38	0.011	0.930	0.195	<.0001	ns	ns	ns
Urea	2.65	3.12	0.158	2.83	2.93	0.158	2.60	2.91	3.13	0.134	0.003	0.370	0.003	ns	0.003	ns

^a^Interactions are described in the text when significant

*ns* not significant

There was an interaction between FO and period for plasma IGF-1 concentration (*P* = 0.057), whereby no effect of FO supplementation was observed in the pre-weaning period, while during the peri-weaning period (*P* = 0.068) and post-weaning period (*P* = 0.05) FO supplemented calves tended to have lower IGF-1 concentrations (peri-weaning: 40.04 vs 84.05 ng/mL, SED = 18.043; post-weaning: 90.81 vs 150.00 ng/mL; SED = 18.20 for FO+ vs FO–, respectively). We also detected an interaction between GL and period for plasma IGF-1 concentration, manifested as no effect of GL supplementation in the pre-weaning or peri-weaning periods, while during the post-weaning period, GL supplemented calves had lower (*P* = 0.023) IGF-1 concentrations (82.64 vs 158.16 ng/mL; SED = 18.2, for GL+ vs GL–). There was a strong tendency towards an interaction between GL and period for glucose concentration (*P* = 0.058) whereby GL supplementation in the pre-weaning period had no effect on glucose concentration (3.69 vs 3.86 mmol/L; SED = 0.178 for GL+ vs GL–), while during the peri-weaning and post-weaning periods GL supplemented calves had lower glucose concentrations than non GL supplemented calves (peri-weaning 3.28 vs 3.85 mmol/L; SED = 0.178 & post-weaning 3.68 vs 4.26 mmol/L; SED = 0.178 for GL+ vs GL–). There was also an interaction between FO and period for plasma urea concentration (*P* = 0.003), manifested as no effect of FO supplementation in the pre-weaning or the post-weaning period while during the peri-weaning period FO supplemented calves had greater (*P* = 0.001) plasma urea concentrations (peri-weaning: 3.33 vs 2.50 mmol/L; SED = 0.137, for FO+ vs FO–). No further two-way interactions between either of FO, GL or period were detected for plasma, metabolic hormones or metabolites.

Mean insulin concentrations in all calves were lower (*P* > 0.001) in the peri-weaning period than in the pre- and post-weaning periods, while mean concentrations of IGF-1 were greater (*P* < 0.01) in all calves during the post-weaning than in pre- and peri-weaning periods. Calves supplemented with FO had a lower mean (*P* < 0.001) plasma glucose concentration than un-supplemented calves. Glucose concentrations in all calves were lowest in the peri-weaning period and greatest in the post-weaning period (*P* = 0.0001). Mean concentrations of βHBA were greater as calf age increased throughout the experiment.

### Blood haematology variables

The effect of dietary supplementation with FO and GL on a number of haematological indicators of immune function is summarised in Table 8. No three way FO × GL × period interactions were observed for any of the haematology variables presented. An interaction between FO supplementation and period was detected for haptoglobin concentration, where no effect of FO was observed in either the pre- or post-weaning periods while during the peri-weaning period, FO supplemented calves tended to have lower (*P* = 0.053) haptoglobin concentrations (Peri: 0.22 vs 0.30 mg/mL; SED = 0.035 for FO+ vs FO–). The concentration of haptoglobin was greater (*P* = 0.034) in calves supplemented with GL compared to their un-supplemented contemporaries. There was no effect of either FO or GL detected on the numbers of white blood cells or red blood cells, or percentages of neutrophils, monocytes or leukocytes (*P* > 0.05). No difference in lymphocyte percentage was observed during any of the three periods examined for FO supplemented or un-supplemented calves; however GL supplemented calves had a lower percentage of lymphocytes (*P* = 0.027) than un-supplemented calves throughout the study. Mean serum osmolality was greater in GL supplemented calves (6.20 vs 6.00; SED = 0.086; *P* = 0.021 for GL+ vs GL–). No effect of FO supplementation on serum osmolality was detected (*P* > 0.05). No effect of FO supplementation on *in vitro* PHA or Con A stimulated IFN-γ production was shown, however GL supplemented calves produced lower levels of PHA stimulated IFN-γ (*P* = 0.019) and Con A stimulated IFN-γ (*P* = 0.012) throughout the experimental period than their un-supplemented contemporaries. Haptoglobin concentrations were greater (*P* = 0.001) and white blood cell numbers were lower (*P* = 0.005) in all calves in the pre-weaning period than in the peri and post-weaning periods. Neutrophil percentages were also greater (*P* = 0.009) and monocytes lower (*P* = 0.01) during the post-weaning period than during the pre- and peri-weaning periods. Serum osmolality was found to be lower (*P* = 0.001) during the pre-weaning period than the peri and post-weaning periods. We also observed an effect of period on in vitro PHA and Con A stimulated IFN-γ production, which declined with increasing age (*P* < 0.0001 and *P* = 0.009, respectively).

**Table 8:** Effect of FO and GL supplementation in calf milk replacer on haematology variables

	Fish Oil (FO)			β-Glucans (GL)			Period (P) relative to weaning				*P*-value^e^		
	–	+	SED	–	+	SED	Pre	Peri	Post	SED	FO	GL	P
Haptoglobin, mg/mL	0.28	0.26	0.024	0.25	0.30	0.024	0.34	0.26	0.22	0.022	0.057	0.034	<.0001
WBC^a^, × 10^3^ cells/μL	11.2	9.4	1.28	10.6	10.0	0.54	9.8^A^	10.5^B^	10.6^B^	0.28	0.185	0.272	0.004
RBC^b^, × 10^6^ cells/μL	23.2	23.0	1.14	23.1	23.1	0.44	23.5^A^	22.6^B^	23.2^A^	0.17	0.940	0.927	<.0001
Neutrophils, %	29.1	29.0	1.81	27.5	30.6	1.81	27.7^A^	28.4^A^	31.1^B^	1.31	0.913	0.149	0.009
Lymphocytes, %	56.5	57.5	1.92	59.2	54.8	1.92	58.2	56.6	56.3	1.11	0.568	0.027	0.114
Monocytes, %	10.4	9.9	0.58	9.9	10.4	0.58	10.3^A^	10.7^A^	9.5^B^	0.33	0.444	0.345	0.001
Leukocyte, %	0.70	0.86	0.138	0.74	0.83	0.050	0.79^A^	0.84^AB^	0.72^AB^	0.044	0.379	0.427	0.001
Osmolality	6.09	6.12	0.086	6.00	6.20	0.086	5.77^A^	6.30^B^	6.23^B^	0.065	0.716	0.021	<.0001
IFN- γ PHA^c^	0.61	0.49	0.074	0.64	0.47	0.074	0.71^A^	0.54^B^	0.41^C^	0.055	0.162	0.019	<.0001
IFN- γ Con A^d^	1.11	1.02	0.072	1.16	0.97	0.072	1.17^A^	1.04^B^	0.99^AB^	0.047	0.234	0.012	0.009

^a^White blood cells

^b^Red blood cells

^c^IFN- γ PHA = Lymphocyte interferon gamma production in response to phytohaemagglutinin A expressed as absorbance at 450 nm

^d^IFN- γ Con A = Lymphocyte interferon gamma production in response to concanavalin A expressed as absorbance at 450 nm

^e^There were no interactions between the main effects detected for haematology variables with the exception of a FO × P interaction for haptoglobin where during the peri-weaning period there was a strong tendency (*P* = 0.053) for FO supplemented calves to have lower haptoglobin concentrations compared with un-supplemented calves (Peri-weaning: 0.22 vs 0.30 mg/mL; SED = 0.035) for FO+ vs FO–), however no differences were detected in either the pre- or post-weaning periods

^A-C^Means within a row with different superscripts differ significantly (*P* < 0.05)

## Discussion

### Feed intake and performance

Nutrition and performance during early life in dairy calves has been reported to play an important role in lifetime performance. Greater ADG pre-weaning is associated with increases in first lactation milk yield in heifers [27], and greater 25-month slaughter weights in Friesian bull calves [28]. Overall, the rates of ADG observed in the present study were comparable to HF bull calves in a recent experiment from our lab which compared pre-weaning rearing regimes for HF and Jersey bull calves at differing planes of nutrition [29]. Calves that received FO had lower ADG to weaning than non FO supplemented calves, which was likely due to the reduced intake of concentrates in the pre-weaning period in calves offered FO. A recent experiment by Ghasemi et al. [30] also showed that voluntary starter concentrate intake, and subsequently ADG, was significantly lower in pre-weaned Holstein calves offered starter containing a 3% fat blend of FO, soya oil and palm fat, compared to un-supplemented controls. During the post-weaning period, calves supplemented with GL had a lower ADG than non GL supplemented calves, which was also probably caused by the reduced intake of concentrates in GL supplemented calves during this period. Additionally, it should be acknowledged that we did not measure DMI of the small quantity of hay offered to each group, however, the observed trends and differences in concentrate intake between groups are likely to be the main factor affecting the varying growth rates observed. Voluntary intake of ryegrass hay by dairy calves was shown in a previous study to average just 46 g DM/d over the pre-weaning period [31], and if we assume similar levels were consumed here it would only equate to approx. 3% of the total DMI. It is also possible that the soya oil (high in linoleic acid, an *n*-6 PUFA) fed to non-FO supplemented calves may have enhanced their performance. Garcia et al. [32] reported improved growth, performance and immune responses in calves consuming 3–5 g/d of linoleic acid and 0.3–0.6 g/d of α-linolenic acid, whilst Ghasemi et al. [30] also reported a tendency towards greater pre-weaning ADG in calves supplemented with soya oil in starter compared to control calves. The soya oil was used in the current study to ensure all diets were iso-lipidic, so as to avoid performance comparisons being confounded by differing dietary energy densities. However, irrespective of the effects of supplementation with either FO or GL on immune function, previous work suggests that the lower ADG observed here in calves offered either of these supplements during the pre and post-weaning period, if extrapolated to dairy heifer calves, may result in reduced milk yield, for at least their first lactation [27, 33].

The crude protein and lipid concentrations of 22.8% and 16.1% present in the base MR used in the current study were comparable with [7], albeit slightly lower in lipid content (16.1% vs 18%). The addition of 40 g of FO per day meant that approximately 7% of mean daily energy intake provided in the MR in the pre-weaning period was in the form of *n*-3 PUFA. The proportion of FO supplemented as a percentage of total DMI would have declined as the experiment progressed due to increasing intake of concentrate. The suppressive effect of FO on DMI intake observed in the current study is well described for more mature cattle by both ourselves [18] and other authors, and was also reported in concentrate starter intake of pre-weaned calves [30]. This effect may also be related to the relatively high supplementation level of FO used here, however all calves not supplemented with FO also consumed an equivalent amount of soya oil, and no suppressive impact on concentrate DMI was apparent. Ballou and DePeters [7], did not offer any calf starter in their experiment which involved supplementation of MR with *n-*3 fatty acids from FO to Jersey calves at an additional 2% of total DMI, and detected no treatment effects on ADG or efficiencies of gain. Their method of delivery of supplementary FA did differ from ours in that they blended the dietary oils with silica dioxide and added them to MR powder before storage in sealed bags at 4 °C [7]. The significantly greater content of EPA and DHA in plasma FA while calves were consuming FO in our study, gives us confidence in the method and delivery of FO used here, which was directly added to the reconstituted MR at each feeding event. Moreover, a further analysis of plasma FA content approximately 1 month post-weaning did not detect any EPA and DHA in the FO supplemented calves.

As regards the reduced concentrate intake observed in GL supplemented calves, greater DMI of starter in control calves than calves fed GL plus ascorbic acid in MR was also shown in an experiment where calves were subjected to a transport stressor in their first 10 d of life and then monitored for 28 d afterwards [15]. Feed intake in the current study, when expressed as MJ of GE/d, was greater in all un-supplemented calves (in receipt of neither FO nor GL supplements) throughout the course of the experimental period, due to the differences in concentrate intake.

### Feeding behaviour

Optimal artificial-rearing husbandry practices, continuous health monitoring, disease investigation and targeted prevention lead to good dairy calf welfare [34]. Computerised feeding systems by their nature give rise to increased competition for milk between calves [35]. There was no observed difference between any of the treatment groups in the number of visits to the feeder where milk was consumed, however the animals not receiving GL had more unrewarded visits to the feeder than GL supplemented calves. A reduction in unrewarded visits to automated milk feeders has previously been associated with increased illness in group housed calves [36, 37]. However, it has also been suggested that a high rate of unrewarded visits is often an indication of increased hunger, particularly at lower levels of intake [38, 39]. This may signify that GL supplementation had a satisfying effect on hunger levels in the GL supplemented calves, particularly given that these calves also spent less time in the concentrate feeder and had less visits per day where concentrates were consumed than their non GL supplemented counterparts. There was no effect of FO supplementation on the amount of unrewarded visits to the MR feeder (mean 11.76 visits/d). This is lower than previously reported values where HF calves receiving 4.8 L per day of MR had a mean total of 31 unrewarded visits per day [38]. However this may be due to the fact that those calves’ daily milk allowance was divided into a minimum of six portions, double the minimum of three daily portions available to our calves. The reduction in unrewarded visits to the calf feeder observed here in GL supplemented calves may have positive consequences in terms of reducing incidences of cross-suckling, a detrimental practice whereby group housed calves direct non-nutritive sucking towards another calves body, which can cause severe problems such as urine consumption and navel ill [40]. In the current study, we also showed that FO supplemented calves had a faster drinking rate than non FO supplemented calves. This effect was not observed for the GL treatment groups. The drinking speed rates observed in the current study are quite high, with all treatment groups averaging drinking speeds in excess of 1 L/min over the pre-weaning period. Interestingly our data are greater than the maximum calf drinking speed of 1 L/min suggested by Haley et al. [40]. By comparison, our lab observed drinking speeds ranging between 0.87–0.99 L/min in similar HF bull calves [29]. The fact that the FO supplemented calves had a significantly greater drinking speed than non FO supplemented calves indicate that there was no issues with the palatability of the FO mixed in with the MR.

### Calf health

Mean fecal scores did not differ between any of the treatment groups over the course of the experimental period despite the differences in total GEI observed; however post-weaning all calves had greater fecal scores compared to the pre-weaning period. In agreement, Ballou and DePeters [7] also observed no effect of FO supplementation on fecal scores of Jersey calves in the pre-weaning period. Interestingly, the mean and range of fecal scores in our study are substantially greater than those reported by Ballou and DePeters [7] and Quigley et al. [41], who both observed scores ranging from 1.4–1.7 in their respective studies. This is most likely due to interpretive differences between the individuals scoring the faeces in the respective experiments, but may also indicate superior health status in calves used in these two studies. Marginally lower fecal scores were reported in calves supplemented with 1 g/d of GL [16], however we did not detect any differences between the respective GL treatment groups in our study.

Although there were small statistically significant differences in rectal temperatures observed between treatments, these differences are unlikely to be of biological importance given that the mean and range of temperatures for all groups were within the normal range for healthy calves. The significantly greater rectal temperatures observed post-weaning in all calves are most likely related to the increased stress and greater susceptibility to infection and disease that has previously been reported in newly weaned calves [42, 43].

### Blood hormone and metabolite concentrations

It is well documented that greater blood concentrations of IGF-1 are associated with increased rates of growth in neonatal calves [44–46], due to its regulation of both skeletal and muscle development in cattle [47]. Furthermore, elevated levels of IGF-1 have been shown to boost immune function [46]; enhanced T-lymphocyte activity in response to stress-induced raised levels of immunosuppressive glucocorticoids has previously been associated with greater levels of serum IGF-1 [48]. In the current study, plasma IGF-1 concentrations were significantly greater in non FO supplemented calves relative to their FO supplemented contemporaries, during both the peri- and post-weaning periods. Similarly, plasma IGF-1 was greater for non GL supplemented calves in the post-weaning period. The positive association between increased feed intake and IGF-1 secretion is well established in dairy calves [41] and is likely the main mechanism explaining the results observed here. These data are in contrast to an earlier study of ours that reported greater concentrations of IGF-1 with increasing dietary FO levels, albeit in older crossbred heifers [18]. In addition there was a significant effect of period on overall IGF-1 levels across all treatments in the current study, which were lowest in the peri-weaning period and highest in the post-weaning period. It is likely this mainly reflects the reduced levels of intake and performance evident in the immediate post-weaning period, but may also be due to an elevated immune response during this period, resulting in an increased production of pro-inflammatory cytokines [49]. However we did not detect any increase in IFN-γ production in the peri-weaning period; instead IFN-γ production was actually lower than in the pre-weaning period. Other pro-inflammatory cytokines such as Interleukin-8 and tumor necrosis factor-α were not measured in the current study and hence may have been present at elevated levels in the peri-weaning period. Reduced growth rates and plasma IGF-1 concentrations have previously been associated with this incidence [41]. Overall, the range in concentrations of IGF-1 reported here are similar to those observed in earlier studies [44, 45] and slightly lower than those recorded by Quigley et al. [41], although in that particular study the greater IGF-1 plasma concentrations were most likely due to the fact that those calves were on a higher plane of nutrition than the calves in our experiment. In contrast, both Graham et al. [46] and Garcia et al. [32] reported much lower concentrations of serum and plasma IGF-1, respectively, in Holstein dairy calves, in the range of 10–42 ng/mL.

The range of values reported in the current study for plasma insulin and glucose concentrations are comparable with those previously observed in Holstein calves [29, 32, 45]. The increased plasma concentrations of glucose in non FO or GL supplemented calves could indicate more efficient absorption of sugars from the MR through the abomasum in these calves during the pre-weaning stage, given that the majority of dietary nutrients in all animals were obtained from the MR during this period. The increased concentrate intake in non FO or GL supplemented calves is also probably associated with the greater plasma glucose levels in these animals, though recent work by Suarez-Mena et al. [50] indicates blood glucose is an unreliable proxy for starter intake in dairy calves. Regardless of the mechanisms involved, in the current study both glucose and insulin results indicated a better metabolic status in calves not receiving FO or GL. The lower levels of plasma glucose and insulin observed in calves supplemented with FO are also in agreement with an earlier study where reduced levels of serum insulin and glucose in calves consuming FO between 8 and 24 h after an endotoxin challenge were reported [10]. Furthermore, Vargas Rodriguez [51] showed that pre-weaned calves supplemented with two levels of DHA derived from algal oil also had lower plasma glucose concentrations than control calves. In contrast to our results, Garcia et al. [32], did not detect any differences in mean plasma concentrations of the anabolic hormones insulin and IGF-1 between four groups of calves receiving increasing amounts of linoleic and α-linolenic acid. Ultimately the significantly higher glucose and insulin levels in the non FO and non GL supplemented calves most likely relates to the greater feed and sugar intake in these calves. Glucose and glutamine are key energy sources of leukocytes, and indeed it has been suggested that a greater availability of glucose in calves could conceivably improve leukocyte function [52].

The increasing concentrations of plasma βHBA as calves grew older is in agreement with several other published studies [29, 32, 53]. Beta hydroxybutyrate is synthesized by ruminal epithelial cells during absorption of butyric acid, and this process is enhanced by increased concentrate intake, as a result of greater levels of butyric acid being produced by microbial fermentation of sugars. Given that increasing blood βHBA concentration is related to initiation of solid feed intake in young ruminants, and thus an indicator of the state of rumen wall metabolic activity [54], the lack of a treatment effect on plasma βHBA concentrations in the current study would appear to indicate that supplementation with either FO or GL has no impact on initiation of rumen wall metabolic activity. Despite this however, concentrate intake was shown to be greater in non FO or GL supplemented calves post-weaning. It is also possible that intake of hay, which was not quantified, was greater in both the FO and GL supplemented calves during this period, compensating for the lower amount of concentrate available for microbial fermentation in the post-weaning period. Plasma urea concentrations increased as calves moved from the pre-weaning phase through to the post-weaning period. This is in agreement with Quigley et al. [41], who attributed greater plasma urea nitrogen concentrations in post-weaned calves to increased ruminal fermentation of dietary protein and subsequent absorption of ammonia from the rumen. The greater plasma urea concentrations observed in the FO supplemented calves during the peri-weaning period may indicate that the stress associated with weaning caused a reduction in metabolism of rumen degradable protein in calves supplemented with FO, particularly given that the total available dietary protein for fermentation would have already been lower in the FO supplemented calves due to the reduced concentrate intake of this group in the post-weaning period. Published work detailing the effects of FO supplementation on blood urea concentrations immediately after weaning is sparse; however the absence of any FO treatment effect on plasma urea concentration in the pre-weaning period is in agreement with previous work [7]. These authors also observed a number of treatment × time interactions in the first 60 d of life on serum concentrations of non-esterified fatty acids, glucose and triaglycerol, however they advised caution in the interpretation of these interactions due to the presence of confounding clinical signs of disease in calves at various stages throughout their study [7]. It is difficult to definitively ascertain the effects of stress on metabolite profiles immediately post-weaning, due to the confounding effects of dietary adaptation following weaning on metabolic profiles [55].

### Blood haematology

Plasma concentrations of the acute phase protein haptoglobin in cattle have previously been reported to change from negligible levels to increases of 100 fold upon stimulation or infection [42, 56] and are therefore a good indicator of the health status of calves [42]. We observed a deviation in the effects of both dietary treatments on haptoglobin levels, where GL supplementation resulted in greater concentrations of haptoglobin throughout the experimental period, while FO supplementation had no effect on haptoglobin concentration in either the pre- or post-weaning periods, but resulted in a strong trend towards lower concentrations of haptoglobin during the peri-weaning period. The raised levels of haptoglobin in GL supplemented calves may indicate an increased inflammatory response to the stress of weaning in these calves, and inversely the lower circulating concentration of haptoglobin in the calves receiving FO suggests that FO supplementation pre-weaning attenuates the inflammatory response in the days post-weaning [57]. There is a dearth of information in the literature on the effects of FO supplementation on circulating haptoglobin concentrations post-weaning, however Garcia et al. [32] did detect slightly higher concentrations in pre-weaned calves fed a lower amount of essential FA and suggested that this may be the result of a greater immune reaction to inflammation of the small intestine in calves receiving more medium chain saturated FA instead of PUFA. Kim et al. [17] also reported increased levels of serum haptoglobin in calves supplemented with hydrolysed yeast containing GL than control calves in the first 3 d following a vaccine challenge. These authors suggested that production of efficient haptoglobin after a vaccine challenge could have beneficial effects on immune responses against incoming pathogens.

A review by Yun et al. [57], asserted that innate immune conditions of calves are stimulated by weaning stress, resulting in an increase in the expression of acute phase proteins and pro-inflammatory cytokines. This observation is supported by our results showing significantly lower PHA and Con A stimulated IFN-γ production, and higher haptoglobin concentrations in GL supplemented calves, suggesting that these calves may have been more immunologically challenged as a consequence of weaning stress. However, it should be added that the effect of GL supplementation on haptoglobin occurred throughout the study, not just during the peri-weaning phase. No effect of supplementation with seaweed extract containing GL on expression of IFN-γ was shown in an earlier study using pigs [12], however these authors did detect an increase in expression of the chemokine interleukin-8 in GL-supplemented pigs. Interleukin-8 is involved in the recruitment and activation of neutrophils from the blood to the site of infection [12]. Neutrophil percentage was greater in the post-weaning period in all calves in the present study, and did not differ between the pre- and peri-weaning periods. Previous studies have shown elevated levels of neutrophil numbers in the immediate period after weaning, followed by a return to pre-weaning levels within 2 weeks [42, 58]. In contrast, our results showed no difference between neutrophil percentages in the pre and peri-weaning periods and a greater neutrophil percentage in the post-weaning period. This tendency towards delayed onset of peripheral neutrophilia post-weaning, instead of in the immediate aftermath of weaning, was unexpected and may be due to our sampling protocol, which included just two measurements of haematology parameters in the post-weaning period, 14 and 31 d post-weaning. However leukocyte percentage was greater in the peri-weaning period, in agreement with Lynch et al. [55] and indicates that calves were under a heightened level of stress in this period. The seven-day length of the weaning period may also have been a factor affecting the observed pro-inflammatory response, as recent work from our group with both HF and Jersey bull calves has shown a 14-day gradual weaning period resulted in minimal changes to neutrophil and lymphocyte numbers during the weaning period [59, 60]. Stress can be defined as a physiological and behavioural state which is brought about by stress hormones and enables the organism to endure, avoid or recover from an aversive condition [61]. The immune system defends against environmental challenges and stresses [62] and communicates with the brain in order to re-establish homeostasis during the immune response to stressful events [63]. The inflammatory response is initiated early on in order to remove the source of disturbance, to enable the organism to adapt to the new conditions and finally to restore homeostasis [61]. The most common theory relating stress to immune function is that stress suppresses immune function in order to maintain more resources for activities which are more important for survival, and consequently, increases disease susceptibility [61, 62]. However, this theory is becoming out-dated as many recent studies have shown that stress can actually enhance immune function [58, 62, 64, 65]. More recently, Johnston et al. [60] used RNA-Seq technology to examine global changes in the whole blood mRNA transcriptome, between Holstein-Friesian and Jersey calves, in response to gradual weaning. The results of these studies demonstrated that the gradual weaning practiced in these studies was welfare-friendly as it did not induce global differential gene expression in whole blood or evoke a physiological stress response in dairy calves [59]. While monocyte numbers in cattle have previously been shown to be variable and inconclusive as biomarkers of stress [58], we detected a slight reduction in monocyte percentage during the post-weaning period. However, no treatment effects on monocyte percentage were detected in the current study. An earlier study showed increased total monocyte numbers in weaned pigs consuming *L. hyperborea* seaweed extract, which contains water insoluble GL [12]. Previously Ballou and DePeters [7], found small differences in monocyte phagocytosis of a preopsonized *E. coli* between control calves and FO supplemented calves in the pre-weaning period, but determined that these changes were too small to influence host defence. The only white blood cell type measured in our study which showed an effect of treatment was lymphocytes, where GL supplemented calves had a lower lymphocyte percentage. O’Loughlin et al. [58] attributed a reduction in lymphocyte numbers 2 d after weaning to the trafficking of lymphocytes from general circulation to tissues and organs at risk of infection, an effect that has previously been reported elsewhere [42]. Taken together with the augmented measures of haptoglobin and IFN-γ in calves consuming GL, this indicates a change in immune function due to GL supplementation, particularly around weaning when stress levels are elevated. Strengthening this theory, the greater levels of serum osmolality in GL supplemented calves may be an indicator of increased incidence of diarrhoea in these animals, which may help to explain the tendency towards marginally higher fecal scores in GL supplemented calves. T-lymphocytes from the FO and GL supplemented calves produced numerically lower amounts of IFN-γ when stimulated by Con A and PHA; however only the GL supplemented calves reached statistical significance. Greater mean production of IFN-γ together with constant or decreased production of the chemokine interleukin-4 signifies an improved ability to switch to a T helper-1 response [66]. As neonates are born with a bias towards T helper-2 against T helper-1 response cells, it can cause an insufficient response to infectious agents [32]. Whilst we did not measure production of interleukin-4 in the current study, the decreased production of IFN-γ observed in GL supplemented calves may potentially indicate reduced cell mediated and humoral immunity in these calves. There was no effect of FO supplementation on any of the haematology variables shown in Table 7 other than haptoglobin. Ballou and DePeters [7] reported no effect of FO supplementation on white blood cell counts and haematocrit percentages in the first 60 d of life. In agreement, we did not detect any effect of FO supplementation on white or red blood cell counts; despite observing numerically lower white blood cell counts in the FO supplemented calves, these differences failed to reach statistical significance. Garcia et al. [32] observed a linear trend towards decreased concentrations of red blood cells in pre-weaned calves receiving increasing amounts of essential FA, and hypothesized that this was related to a reduced incidence of diarrhoea in calves receiving more essential FA; greater haematocrit percentages and red blood cell concentrations have previously been linked to increased dehydration caused by more intense severity of diarrhoea.

## Conclusions

Supplementation of calf MR with either FO or GL failed to evoke any clear positive effects on either performance or indices of immune function, during both the pre- and post-weaning period. Indeed there was evidence that both supplements may have counterproductive effects for calves, as evidenced by the reduced DMI of concentrates and lower weaning, turnout and end weights in GL and FO supplemented calves. Most of the metabolic and immune measures reported here appeared to reflect treatment effects on feed intake and animal performance. Furthermore, the recent industry shift towards feeding a higher plane of nutrition to accelerate growth early in the pre-weaning period would indicate that supplementation with these levels of GL and FO, at least in the manner used in our study, would be impractical for producers. This study does offer a novel insight into disparities in feeding behaviour throughout the first 90 d of life in calves supplemented with FO and GL pre-weaning. The observed haematology variables provide further understanding into how the addition of FO and GL to the diet of neonatal calves can augment immune function in both the pre and post-weaning periods. Ultimately however, the inferior performance data observed in FO and GL supplemented calves suggests that any future work involving FO or GL should be done at lower levels of supplementation. Furthermore, any future studies should focus on identifying in more detail the immune function indicators not measured here, such as neutrophil oxidative burst, fibrinogen concentrations, stress related hormone concentrations and expression of pro and anti-inflammatory cytokines, whilst simultaneously ensuring calf health, performance and starter intake is not depressed by either treatment.

## Abbreviations

ADG: Average daily gain
Con A: Concanavalin A
CON: Control
DHA: Docosahexaenoic acid
EPA: Eicosapentaenoic acid
FA: Fatty acid
FO: Fish oil
FOGL: Fish oil & β-glucans
GE: Gross energy
GEI: Gross energy intake
GL: β-glucans
HF: Holstein-Friesian
IFN-γ: interferon gamma
IGF-1: Insulin-like growth factor 1
MR: Milk replacer
*n*-3: Omega 3
PHA: Phytohaemagglutinin
PUFA: Poly-unsaturated fatty acids
βHBA: β-hydroxybutyrate

## References

[1] Raboisson D, Delor F, Cahuzac E, Gendre C, Sans P, Allaire G (2013). Perinatal, neonatal, and rearing period mortality of dairy calves and replacement heifers in France. J Dairy Sci.

[2] Henderson LF, Miglior A, Sewalem A, Kelton D, Robinson A, Leslie KE (2011). Estimation of genetic parameters for measures of calf survival in a population of Holstein heifer calves from a heifer-raising facility in New York State. J Dairy Sci.

[3] O’Doherty JV, Pierce KM, Kenny DA. Fermentable fibre and gut health in non- and pre-ruminants. In: Garnsworthy PC, Wiseman J, editors. Recent advances in animal nutrition: Nottingham University Press; 2005. p. 103–28.

[4] Leonard SG, Sweeney T, Bahar B, Lynch BP, O’Doherty JV (2010). Effect of maternal fish oil and seaweed extract supplementation on colostrum and milk composition, humoral immune response, and performance of suckled piglets. J Anim Sci.

[5] Calder PC (1998). Dietary fatty acids and the immune system. Nutr Rev.

[6] Calder PC (2015). Marine omega-3 fatty acids and inflammatory processes: effects, mechanisms and clinical relevance. Biochim Biophys Acta.

[7] Ballou MA, DePeters EJ (2008). Supplementing milk replacer with omega-3 fatty acids from fish oil on immunocompetence and health of Jersey calves. J Dairy Sci.

[8] Muturi KN, Scaife JR, Lomax MA, Jackson F, Huntley J, Coop RL (2005). The effect of dietary polyunsaturated fatty acids (PUFA) on infection with the nematodes Ostertagia ostertagi and Cooperia oncophora in calves. Vet parasitology.

[9] Meydani SN, Endres S, Woods MM, Goldin BR, Soo C, Morrill-Labrode A (1991). Oral (n-3) fatty acid supplementation suppresses cytokine production and lymphocyte: comparison between young and older women. J Nutr.

[10] Ballou MA, Cruz GD, Pittroff W, Keisler DH, DePeters EJ (2008). Modifying the acute phase response of Jersey calves by supplementing milk replacer with omega-3 fatty acids from fish oil. J Dairy Sci.

[11] Sweeney T, Collins B, Reilly P, Pierce KM, Ryan M, O’Doherty JV (2012). Effect of purified β-glucans derived from *Laminaria digitata*, *Laminaria hyperborea* and *Saccharomyces cerevisiae* on piglet performance, selected bacterial populations, volatile fatty acids and pro-inflammatory cytokines in the gastrointestinal tract of pigs. Br J Nutr.

[12] Reilly P, O’Doherty JV, Pierce KM, Callan JJ, O’Sullivan JT, Sweeney T (2008). The effects of seaweed extract inclusion on gut morphology, selected intestinal microbiota, nutrient digestibility, volatile fatty acid concentrations and the immune status of the weaned pig. Animal.

[13] Yun CH, Estrada A, Kessel AV, Park BC, Laarveld B (2003). β-Glucan, extracted from oat, enhances disease resistance against bacterial and parasitic infections. FEMS Immu Med Microbiol.

[14] Brown GD, Gordon S (2005). Immune recognition of fungal β-glucans. Cell Microbiol.

[15] Eicher SD, Wesley IV, Sharma VK, Johnson TR (2010). Yeast cell-wall products containing β-glucan plus ascorbic acid affect neonatal *Bos taurus* calf leukocyte and growth after a transport stressor. J Anim Sci.

[16] Davis EM (2018). The impacts of various milk replacer supplements on the health and performance of high-risk dairy calves.

[17] Kim MH, Seo JK, Yun CH, Kang SJ, Ko JY, Ha JK (2011). Effects of hydrolyzed yeast supplementation in calf starter on immune responses to vaccine challenge in neonatal calves. Animal.

[18] Childs S, Hennessy AA, Sreenan JM, Wathes DC, Cheng Z, Stanton C (2008). Effect of level of dietary n-3 polyunsaturated fatty acid supplementation on systemic and tissue fatty acid concentrations and on selected reproductive variables in cattle. Theriogenology.

[19] Lynch MB, Sweeney T, Callan JJ, O’Sullivan JT, O’Doherty JV (2010). The effect of dietary *Laminaria*-derived laminarin and fucoidan on nutrient digestibility, nitrogen utilisation, intestinal microflora and volatile fatty acid concentration in pigs. J Sci Food Agric.

[20] Brooks HW, Michell AR, Wagstaff AJ, White DG (1996). Fallability of faecal consistency as a criterion of success in the evaluation of oral fluid therapy for calf diarrhoea. Br Vet J.

[21] Eckersall PD, Duthie S, Safi S, Moffatt D, Horadagoda NU, Doyle S (1999). An automated biochemical assay for haptoglobin: prevention of interference from albumin. Comp Haem Inter.

[22] Wood PR, Corner LA, Plackett P (1990). Development of a simple, rapid in vitro cellular assay for bovine tuberculosis based on the production of gamma interferon. Res Vet Sci.

[23] Rothel JS, Jones SL, Corner LA, Cox JC, Wood PR (1990). A sandwich enzyme immunoassay for bovine interferongamma and its use for the detection of tuberculosis in cattle. Aust Vet J.

[24] Christie W (1982). A simple procedure for rapid transmethylation of glycerolipids and cholesteryl esters. J Lipid Res.

[25] Van Soest PJ, Robertson JB, Lewis BA (1991). Methods for dietary fiber, neutral detergent fiber and non starch polysaccharides in relation to animal nutrition. J Dairy Sci.

[26] Sweeney RA (1989). Generic combustion method for determination of crude protein in feeds: collaborative study. J Assoc Off Anal Chem.

[27] Soberon F, Van Amburgh ME (2013). The effect of nutrient intake from milk or milk replacer of pre-weaned dairy calves on lactation yield as adults: a meta-analysis of current data. J Anim Sci.

[28] Fallon RJ, Harte FJ (1986). Effect of giving three different levels of milk replacer on calf performance. Irish J Agr Res.

[29] Byrne CJ, Fair S, English AM, Johnston D, Lonergan P, Kenny DA (2017). Effect of milk replacer and concentrate intake on growth rate, feeding behaviour and systemic metabolite concentrations of pre-weaned bull calves of two dairy breeds. Animal.

[30] Ghasemi E, Azad-Shahraki M, Khorvash M (2017). Effect of different fat supplements on performance of dairy calves during cold season. J Dairy Sci.

[31] Castells L, Bach A, Araujo G, Montoro C, Terré M (2012). Effect of different forage sources on performance and feeding behavior of Holstein calves. J Dairy Sci.

[32] Garcia M, Shin JH, Schlaefli A, Greco LF, Maunsell FP, Thatcher WW (2015). Increasing intake of essential fatty acids from milk replacer benefits performance, immune responses, and health of preweaned Holstein calves. J Dairy Sci.

[33] Soberon F, Raffrenato E, Everett RW, Van Amburgh ME (2012). Preweaning milk replacer intake and effects on long term productivity of dairy calves. J Dairy Sci.

[34] Hulbert LE, Moisa SJ (2016). Stress, immunity, and the management of calves. J Dairy Sci.

[35] Jensen MB, Weary D (2013). Group housing and milk feeding of dairy calves. Proc. 25^th^ Western Canadian dairy seminar - advances in dairy. Technology.

[36] Svensson C, Jensen MB (2007). Short communication: identification of diseased calves by use of data from automatic milk feeders. J Dairy Sci.

[37] Borderas TF, de Passille AMB, Rushen J (2009). Feeding behavior of calves fed small or large amounts of milk. J Dairy Sci.

[38] Jensen MB, Holm L (2003). The effect of milk flow rate and milk allowance on feeding behaviour in dairy calves fed by computer controlled milk feeders. Appl Anim Behav Sci.

[39] Jensen MB (2006). Computer-controlled milk feeding of group-housed calves: the effect of milk allowance and weaning type. J Dairy Sci.

[40] Haley DB, Rushen J, Duncan IHJ, Widowski TM, de Passille AM (1998). Effects of resistance to milk flow and the provision of hay on nonnutritive sucking by dairy calves. J Dairy Sci.

[41] Quigley JD, Wolfe TA, Elsasser TH (2006). Effects of additional milk replacer feeding on calf health, growth, and selected blood metabolites in calves. J Dairy Sci.

[42] Hickey MC, Drennan M, Earley B (2003). The effect of abrupt weaning of suckler calves on the plasma concentrations of cortisol, catecholamines, leukocytes, acute-phase proteins and in vitro interferon- gamma production. J Anim Sci.

[43] Lynch EM, McGee M, Doyle S, Earley B (2011). Effect of post-weaning management practices on physiological and immunological responses of weaned beef calves. Irish J Agric Food Res.

[44] Brown EG, VandeHaar MJ, Daniels KM, Liesman JS, Chapin LT, Keisler DH (2005). Effect of increasing energy and protein intake on body growth and carcass composition of heifer calves. J Dairy Sci.

[45] Daniels KM, Hill SR, Knowlton KF, James RE, McGilliard ML, Akers RM (2008). Effects of milk replacer composition on selected blood metabolites and hormones in preweaned Holstein heifers. J Dairy Sci.

[46] Graham TW, Breher JE, Farver TB, Cullor JS, Kehrli ME, Oberbauer AM (2010). Biological markers of neonatal calf performance: the relationship of insulin-like growth factor-I, zinc, and copper to poor neonatal growth. J Anim Sci.

[47] Bass J, Oldham J, Sharma M, Kambadur R (1999). Growth factors controlling muscle development. Domest Anim Endocrinol.

[48] Dorshkind K, Horseman ND (2001). Anterior pituitary hormones, stress, and immune system homeostasis. Bioessays.

[49] Brickell JS, McGowan MM, Wathes DC (2009). Effect of management factors and blood metabolites during the rearing period on growth in dairy heifers on UK farms. Dom Anim Endocrinology.

[50] Suarez-Mena FX, Hu W, Dennis TS, Hill TM, Schlotterbeck RL (2017). β-Hydroxybutyrate (BHB) and glucose concentrations in the blood of dairy calves as influenced by age, vaccination stress, weaning, and starter intake including evaluation of BHB and glucose markers of starter intake. J Dairy Sci.

[51] Vargas-Rodriguez CF. Effect of dietary fatty acids and other nutritional supplements on biological processes in dairy cows. USA: Kansas State University; 2016. PhD thesis.

[52] Ballou MA (2012). Immune responses of Holstein and Jersey calves during the pre- and immediate post-weaned periods when fed varying planes of milk replacer. J Dairy Sci.

[53] Khan MA, Lee HJ, Lee WS, Kim HS, Ki KS, Hur TY (2007). Structural growth, rumen development, and metabolic and immune responses of Holstein male calves fed milk through step-down and conventional methods. J Dairy Sci.

[54] Baldwin RLVI, McLeod KR, Klotz JL, Heitmann RN (2004). Rumen development, intestinal growth and hepatic metabolism in the pre- and post-weaning ruminant. J Dairy Sci.

[55] Lynch EM, McGee M, Doyle S, Earley B (2012). Effect of preweaning concentrate supplementation on peripheral distribution of leukocytes, functional activity of neutrophils, acute phase protein and behavioural responses of abruptly weaned and housed beef calves. BMC Vet Res.

[56] Earley B, Crowe MA (2002). Effects of ketoprofen alone or in combination with local anesthesia during the castration of bull calves on plasma cortisol, immunological, and inflammatory responses. J Anim Sci.

[57] Yun CH, Wynn P, Ha JK (2014). Stress, acute phase proteins and immune modulation in calves. Anim Prod Sci.

[58] O'Loughlin A, McGee M, Waters S, Doyle S, Earley B (2011). Examination of the bovine leukocyte environment using immunogenetic biomarkers to assess immunocompetence following exposure to weaning stress. BMC Vet Res.

[59] Johnston D, Kenny DA, Kelly AK, McCabe MS, McGee M, Waters SM (2016). Characterisation of haematological profiles and whole blood relative gene expression levels in Holstein-Friesian and Jersey bull calves undergoing gradual weaning. Animal.

[60] Johnston D, Earley B, Cormican P, Kenny DA, McCabe MS, Kelly AK (2016). Characterisation of the whole blood mRNA transcriptome in Holstein-Friesian and Jersey calves in response to gradual weaning. PLoS One.

[61] Martin LB (2009). Stress and immunity in wild vertebrates: timing is everything. Gen Comp Endocrinol.

[62] Salak-Johnson JL, McGlone JJ (2007). Making sense of apparently conflicting data: stress and immunity in swine and cattle. J Anim Sci.

[63] Elenkov I, Iezzoni D, Daly A, Harris A, Chrousos G (2005). Cytokine dysregulation, inflammation and well-being. Neuroimmunomodulation.

[64] O'Loughlin A, Lynn D, McGee M, Doyle S, McCabe M, Earley B (2012). Transcriptomic analysis of the stress response to weaning at housing in bovine leukocytes using RNA-seq technology. BMC Genomics.

[65] Carroll JA, Burdick Sanchez NC, Bill E (2014). Kunkle interdisciplinary beef symposium: overlapping physiological responses and endocrine biomarkers that are indicative of stress responsiveness and immune function in beef cattle. J Anim Sci.

[66] Chase CCL, Hurley DJ, Reber AJ (2008). Neonatal immune development in the calf and its impact on vaccine response. Vet Clin North Am Food Anim Pract.

